# Enlargement of the Journal and Library of Dental Science

**Published:** 1846-09

**Authors:** 


*Enlargement of the Journal and Library of Dental Science.*—Wishing to make this publication as worthy of the patronage of the dental profession as possible, the conductors have, from year to year, gradually enlarged it. The sixth volume contains sixty-four pages more matter than the fifth, and it is our purpose, if the subscribers will enable us to do it, by paying their subscriptions promptly, and assisting to extend its circulation, to give 144 pages more in the present than is contained in the last volume. Each number will then contain two hundred pages, making, at the end of the year, two volumes of four hundred pages each. It is also our intention to have it printed on better paper; but, in order to meet the additional expense which we shall incur in its publication, it will be absolutely necessary that the subscriptions be *promptly* paid, and that *all* who are in arrears remit *immediately* the amount of their indebtedness. Without this co-operation of the subscribers, we shall be unable to carry out our designs. We mention this now, believing that every subscriber, inasmuch as it is so much to his interest to do so, will cheerfully and at once respond to this appeal, and it is the more necessary that every one should do so, as our subscription is very limited.

We ought to have in the United States four times the number of subscribers we have. There are some fifteen or eighteen hundred dentists in this country, and we have not more than two hundred and seventy-five or three hundred subscribers from among them. We have been informed that there are nearly two hundred dentists in the city of New York alone, and from this number there are only some thirteen or fourteen who take the Journal. Philadelphia does a little better. We have twenty-one or two subscribers there out of about one hundred dentists. Baltimore does better than either Philadelphia or New York. There are about thirty-five dentists here, and out of this number sixteen take the Journal. What a commentary upon the intelligence of the dental profession generally in the United States! And, on the other hand, when we take into consideration that in no country in the world has our art been more zealously cultivated, or brought to a higher state of perfection than in this, what an eulogium upon the few who have not only prevented it from sinking into utter degradation, but have been instrumental in securing for it such an enviable position! Yes, although the greater number, by far, of those engaged in the practice of dentistry in this country are ignorant and illiterate men, totally unqualified for the calling, he number of scientific, skilful practitioners is rapidly increasing, and we oelieve the day is not distant, when it will be required of all exercising the duties of this branch of the curative art, to possess as high claims to skill, as it now is of the general practitioner. The dawning of this day is already beginning to be seen in the union of effort that is being made by very many of the best informed members of the profession, to elevate the standard of qualification. Let not, then, the efforts of those who are engaged in this noble work relax, but rather let the good which has already resulted from their labors incite them to redouble their exertions for the accomplishment of an object in which their own reputation, as well as the good of mankind, are involved.

But to return from this digression: Some complain of the price of subscription to the Journal, as being too high, compared with that charged for kindred publications, without taking into consideration the limited number of subscribers, supposing that it could be furnished to three or four hundred at as low a rate as it could be furnished to a thousand or fifteen hundred. The increase in its size and in the value of the paper will, in a measure, do away with this objection. There is, however, a fact which those who have made it, seem to have entirely overlooked. It is this: the works in the library part alone could not, in any other way, be procured for three times the subscription price of the Journal, and some of them are publications which were before out of print, and could not be had at any price; others, again, were in a foreign language, and wholly unintelligible to most of our readers, which we have had translated at considerable expense. But few, however, have objected to the price of the Journal, and some, appreciating, in its proper light, the value of such a publication, have insisted upon paying double the amount charged for it.

As further evidence of a disposition on our part to do all we can to enhance the value of the publication, and make it acceptable to the profession, we commence, in the present number, the publication of the work of Desirabode, which we noticed in our last, as having been just received. It will occupy the whole of the library part of four and perhaps of five numbers, and those who wish to procure this celebrated treatise should lose no time in subscribing for the Journal.

We want one hundred more names to our subscription list, and half of this number we expect to obtain in England, during the coming year, where I believe we already have about one hundred, and if those who take the Journal in this country would make a little effort, we have no doubt that we should soon have the other fifty.—*Balt. Ed.*

